# Effects of monthly evaluations on the rates of irrational antimicrobial prescription in the outpatient and emergency departments at Ningbo No. 6 Hospital, Ningbo, China

**DOI:** 10.1186/s40001-022-00728-6

**Published:** 2022-06-22

**Authors:** Qiong Yang, Fangfang Yuan, Li Li, Jianfeng Jin, Junhong He

**Affiliations:** 1 Department of Pharmacy, Ningbo No. 6 Hospital, Ningbo, China; 2 Department of Rheumatism and Immunology, Ningbo No. 6 Hospital, Ningbo, China

**Keywords:** Antibiotic usage, Antibiotic resistance, Antibiotic prescription rate, Monthly evaluation

## Abstract

**Background:**

Antibiotic resistance is a major global public health problem. The primary cause of antibiotic resistance is inappropriate antibiotic use. In this study, we aimed to verify whether the monthly evaluation of antibiotic prescription improves clinical antibiotic use in outpatient and emergency departments.

**Methods:**

A minimum of 25% of the prescriptions for antibacterial drugs were randomly selected at the outpatient and emergency departments to enter the monthly evaluation system from July 2016 to June 2019. We analysed the rate of irrational prescription of antibiotics, proportion of the use of antibiotics, and consistency between the evaluation and expert groups after implementing the monthly assessment to validate the role of monthly evaluations.

**Results:**

After 3 years of monthly evaluations of antibiotic prescriptions, the utilisation rate of single antibiotics in the outpatient and emergency departments was found to increase each year. Each year, a decreasing trend was observed for the irrational use of antibiotics, whereas the proportion of antibiotics to the total drugs prescribed gradually decreased in the same period. In addition, the consistency of prescription evaluation results between the evaluation and expert groups increased continuously.

**Conclusions:**

Monthly evaluation of antibiotic prescriptions is an effective management tool for the rational use of antibiotics in clinical practice. This practice could help reduce the combinative use of antibiotics, rate of irrational antibiotic prescription, and antibiotic use ratio, and play an important role in safe clinical drug use.

## Background

Since the 1940s, an increasing number of antibiotics have been developed for human use. Antibiotics have saved countless lives in clinical practice, and they are among the most commonly prescribed drugs in human medicine [1]. However, with the increasing use of antibiotics, resistance has significantly increased worldwide [2]. Antibiotic resistance leads to treatment failures, prolonged hospital stays, and increased medical expenses and mortality rate [3]. In 2011, the theme for the World Health Day was ‘Combat Antimicrobial Resistance’; indeed, the World Health Organization called for global attention to the problem of antibiotic resistance [4, 5]. Ten years later, antibiotic resistance remains a major public health problem. More than 2.8 million people are infected with drug-resistant microbes each year in the United States alone, resulting in more than 35,000 deaths and hospitalisations. The economic and human costs associated with these infections are enormous. By 2050, the number of deaths from drug-resistant infections is expected to increase to 10 million a year, surpassing diabetes, heart disease, and cancer as the leading causes of human deaths [6].

The primary cause of antibiotic resistance is the inappropriate use of antibiotics. Accordingly, it is necessary to reduce the excessive rate of antibiotic prescription and improve the use of antimicrobials to curb resistance [7]. Performing audits and providing feedback on antibiotic prescriptions have been shown to effectively control antibiotic use and influence clinical outcomes. For example, prospective audits with interventions and feedback can significantly reduce the total number and improve the quality of antibiotic prescriptions [8, 9]. Integrating prescription restrictions, approval systems, and computer decision support into the electronic prescription process is an effective measure for improving antibiotic resistance [10–12]. Although prescription restrictions, approval systems, and computerised decision support are automated systems, they still require up-front human setup to function effectively. Evaluation criteria for automated systems are obtained from experienced pharmacists. In addition, with in-depth disease and drug research, the use of drugs is becoming more complicated, and the above automatic systems still require regular evaluation and optimisation by pharmacists. Therefore, the measures mentioned above were designed to improve the prescription process for antibiotics, but they are limited by the ability of individual reviewers. In addition, the effect of periodic retrospective analyses of antibiotic prescriptions on antibiotic use remains largely unexplored. We believe that professional evaluation teams rather than individual reviewers can have a more significant effect on antibiotic management. Unfortunately, periodic sampling evaluation of antibacterial prescriptions has rarely been reported. In this study, we established a monthly evaluation system, combined with an electronic prescription system, and verified whether the system could improve the rational prescription of antibacterial drugs. This innovative system involved three rounds of evaluation and publicity and served as an important supplement to prospective prescription reviews by improving the professionalism of physicians and evaluators.

## Methods

### Data source

A total of 58 749 antibiotic prescriptions from the outpatient and emergency departments were randomly selected from the Ningbo No. 6  Hospital from July 2016 to June 2019.

### Basis for assessment

The appropriateness of prescription was evaluated based on the following documents and database.

- Drug description
- *Prescribing point review management practices (Trial),* 2010, China [13]
- *Scheme of special remediation activity on the clinical application of antimicrobial drugs,* 2012, China [14]
- *Guidelines for clinical use of antimicrobial agents,* 2015, China [15]
- Clinical guidelines
- UpToDate database (Wolters Kluwer, Alphen aan den Rijn, The Netherlands).

### Sampling method

We first enabled the IPharmacare Drug Management System (IPharmacare, Hangzhou, Zhejiang, China). The IPharmacare Drug Management System is established by IPharmacare (Hangzhou, Zhejiang, China). The system is used for hospital drug management, including drug approval, prescription restrictions, and random sampling of prescriptions. Every month, 25% of the doctors in our hospital with the right to prescribe antibiotics were randomly selected by this system to evaluate the antibiotics prescribed in the outpatient and emergency departments. Among the doctors selected by the system, if a doctor had made more than 50 prescriptions, we drew 50 prescriptions, and if a doctor had made than 50 prescriptions, we evaluated all prescriptions. An additional parameter was that the number of prescriptions should not be less than 25% of the total prescribed in the hospital.

The above selection ratio and quantity are based on *a scheme of special remediation activity for the clinical application of antimicrobial drugs, 2012, China *[14]*.*

### Evaluation method

Prescription evaluation teams were established under the leadership of the Medical Services section and consisted of working groups and expert group members. The evaluation team consisted of six clinical pharmacists who conducted an initial evaluation of randomly selected prescriptions, and the expert group consisted of two chief pharmacists and two chief physicians who conducted a re-evaluation of irrationally prescribed antibiotics. For the initial evaluation, the clinical pharmacists assessed sample prescriptions, and prescriptions of antibiotics prescribed irrationally were recorded using Microsoft Excel (2010) spreadsheets. Irrational prescription types, problem descriptions, medication suggestions, and other parameters were then analysed.

Next, the prescription evaluation team leader re-evaluated any unreasonable prescriptions, which were then publicised on the hospital intranet for 1 week. The prescription evaluation team leader was contacted for any issues, and experts were organised to re-evaluate the problem to obtain the final evaluation result.

The sampling and preliminary evaluation of the previous month were completed before the 15th of each month. The evaluation results were publicised and studied by the entire hospital staff on the 15th of each month. If there were any objections within 7 days, the expert group would start the re-evaluation; otherwise, the evaluation result remained in effect.

### Assessment measures

Unreasonable prescriptions were publicised regularly, and the final result was included in evaluating individual senior professional titles. Every doctor has a benchmark score of 100 each year. For each unqualified prescription, 0.1 point was deducted from the benchmark score. The result of applying for a senior professional position and the income of a doctor were related to the score.

### Statistical analysis

SPSS version 16.0 (IBM, Armonk, NY, USA) was used for data processing, and results with *P* < 0.05 were considered statistically significant. The irrational prescription rates of antibacterial drugs in different years were analysed using the chi-square test.

## Results

### Antibiotic prescriptions in the outpatient and emergency departments

After implementing the monthly evaluation system, the rate of single antibiotic prescriptions in the outpatient departments increased each year from July 2016 to June 2019; the rate of single antibiotic prescriptions in the outpatient and emergency departments increased by 3.53 and 2.99 percentage points, respectively. In addition, the rate of combined prescriptions of two antibiotics decreased with time. Notably, the prescription rate in the emergency department was always higher than that in the outpatient department each year. In 2018 and 2019, the rate of triple antibiotic prescriptions was 0.00% (Table 1). From an economic perspective, the expense of antimicrobial drug prescription decreased from 16.00% of the total drug expense in 2016 to 14.73% in 2019. However, this difference was not statistically significant and may be due to the proportion of emergency antibiotics used (Table 2). These results suggest that the monthly evaluation system potentially helped in controlling the number of antibiotic prescriptions.

**Table 1:** Proportion and features of antibiotic prescriptions in the outpatient and emergency departments in Ningbo No. 6 Hospital, Ningbo, China, from July 2016 to December 2019

A. In the outpatient department				
Parameter	2016	2017	2018	2019
Number of patients	169,676	341,774	353,385	368,923
Number of antibacterial agents used	26,953	56,430	57,615	55,853
Proportion of antibiotic prescriptions (%)	15.88	16.51	16.30	15.14
Utilisation rate of single antibiotic prescriptions (%)	89.70	90.38	90.02	93.23^****^
Utilisation rate of two antibiotic prescriptions (%)	10.23	9.55	9.98	6.77^****^
Utilisation rate of triple antibiotic prescriptions (%)	0.07	0.07	0.00	0.00

B. In emergency department				
Parameter	2016	2017	2018	2019
Number of patients	32,017	64,226	62,886	62,778
Number of antibacterial agents used	15,708	32,540	31,453	32,519
Proportion of antibiotic prescriptions (%)	49.06	50.66	50.02	51.80
Utilisation rate of single antibiotic prescriptions (%)	86.76	85.37	87.45	84.52^***^
Utilisation rate of two antibiotic prescriptions (%)	12.71	14.04	12.54	15.48^****^
Utilisation rate of triple antibiotic prescriptions (%)	0.53	0.59	0.00	0.00

^****^Indicates that the data were statistically significant, *P* < 0.0001; Data for 2016 were obtained from July to December

**Table 2:** Percentage of antibiotic prescriptions in the outpatient and emergency departments in Ningbo No. 6 Hospital, Ningbo, China, from July 2016 to December 2019

Year	Total amount of drugs prescribed (Ten thousand Yuan)	Total amount of antibiotics (Ten thousand Yuan)	Percentage
2016	2318.4	371.01	16.00
2017	4883.52	765.81	15.68
2018	5061.06	741.03	14.64
2019	5058.64	745.5	14.74

Data for 2016 were obtained from July to December

### Rate of irrational antimicrobial drug prescription

An important aspect of the monthly evaluation system was notification to the relevant physician and publication of why the prescription was classified as unreasonable. The effect of this measure was directly reflected in the decrease in the proportion of irrational prescriptions. From July 2016 to June 2019, the rate of irrational prescriptions showed a significant downward trend. The irrationality rate in 2019 decreased by 1.6 percentage points compared with the rate in July–December 2016 (Table 3).

**Table 3:** Rate of irrational antimicrobial drug prescription in Ningbo No. 6 Hospital, Ningbo, China, from July 2016 to December 2019

Year	Total number of prescriptions evaluated	Total number of prescriptions with irrational antibiotic prescription	Percentage
2016	7397	222	3.00
2017	17,908	477	2.67
2018	18,581	427	2.27
2019	18,403	219	1.19^****^

^****^Indicates that the data were statistically significant, *P* < 0.0001. Data for 2016 were obtained from July to December

These irrational prescriptions consist of four categories of problems: irrational antibiotic combined therapy, irrational usage and dosage, medications not matching the diagnosis, and no indication of antibiotics. From July 2016 to June 2019, the main problem of irrational prescription gradually shifted from irrational usage and dosage to no indication of antibiotics (Table 4).

**Table 4:** Proportion and features of irrational antibiotic prescriptions in the outpatient and emergency departments in Ningbo No. 6 Hospital, Ningbo, China, from July 2016 to December 2019

Parameter	2016	2017	2018	2019
Irrational antibiotic combined therapy	9 (4.04%)	37 (7.76%)	22 (5.23%)	76 (37.25%)
Irrational usage and dosage	132 (59.19%)	244 (51.15%)	160 (38.00^)	3 (1.47%)
Medications not matching the diagnosis	25 (11.21%)	61 (12.79%)	94 (22.33%)	1 (0.49%)
No indication of antibiotics	57 (25.56%)	135 (28.30%)	145 (34.34%)	124 (60.78%)
Total	223	477	421	204

### Consistency of prescription evaluation results

Considering the possibility of misevaluation by the evaluation team, a reassessment system and feedback channel were incorporated into the evaluation system. The number of unqualified prescriptions obtained in each expert group reassessment and the number of unqualified prescriptions obtained in the initial evaluations by clinical pharmacists were summarised to determine the consistency of prescription evaluation results for the initial reassessment. The results showed that from 2016 to 2019, the consistency rate between the initial and re-evaluation results increased (Table 5).

**Table 5:** Consistency of prescription evaluation results from the evaluation team and the expert group in Ningbo No. 6 Hospital, Ningbo, China, from July 2016 to December 2019

Year	Total number of irrational prescriptions in preliminary evaluation	Total number of irrational prescriptions in re-evaluation	Consistency (%)
2016	350	222	85.71
2017	601	533	88.69
2018	473	427	90.27
2019	501	480	95.81^***^

Consistency of prescription evaluation (100%) = Total number of irrational prescriptions in re-evaluation/total number of irrational prescriptions in preliminary evaluation × 100. *** indicates that the data were considered statistically significant, *P* < 0.001. Data for 2016 were obtained from July to December

## Discussion

Antibiotics play an important role in modern medicine. The discovery and use of antibiotics have led to medical breakthroughs, such as infection prevention after surgery. Furthermore, antibiotics have made substantial contributions to the control of infectious diseases [16]. As a result, human life expectancy has considerably increased in the second half of the last century. However, the problem of antibiotic resistance is growing rapidly. Resistance to widely used β-lactam antibiotics has developed with increasing frequency. Treatment of ceftriaxone- and fluoroquinolone-resistant gonorrhoeae has become challenging [2]. Vancomycin, a last line of treatment, can no longer completely kill *Enterococcus faecalis* [17]. Although the spread of antibiotic-resistant bacteria threatens human health, research on new antibiotics is slow. Owing to economic reasons, large pharmaceutical companies are turning to the research and development of drugs for chronic diseases [18]. We are now entering the post-antibiotic era. The loss of antibiotics means that common bacterial infections can kill people.

Fortunately, an association between antibiotic use and resistance has been recognised [19]. Prospective review and intervention of antibiotic prescriptions are considered a necessary measure in most healthcare systems. Antibiotic prescription management and monitoring with the participation of clinical pharmacists can effectively reduce the irrational use of antibiotics and financial waste [18, 20, 21]. The use of electronic prescriptions has also improved the effectiveness of antibiotic management. In addition, studies have found that different multi-antibiotic management regimens can achieve better control than a single program [22]. However, it is still necessary to explore other ways to manage antibiotic use.

In this study, we established a monthly evaluation system using random sampling and retrospective analysis of antibiotic prescriptions in the outpatient and emergency departments in a hospital. In contrast to the prospective reviews and interventions by clinical pharmacists, the monthly evaluation system was a response to the execution or review of antibiotic prescriptions. The monthly evaluation system supplements prospective review and intervention systems. Its main function is to compensate for human negligence in prescription review and, more importantly, to optimise the qualified prescription. This was demonstrated by the change in the proportion of antibiotics prescribed throughout this study, particularly the reduced rate of irrational prescription of antibiotics. Although the prescriptions approved by clinical pharmacists were qualified and had good curative effects, there was still room for improvement in the management of antibiotics owing to the differences in the experience and prescribing habits of doctors. Once the prescriptions were evaluated, they were reviewed by the evaluation team leader. Following both these rounds of review, the evaluation errors were minimised. The subsequent publicity of the results was one of the keys to the system. A US study suggested that disclosing institutional assessment scores could eventually change clinical practice [23]. Similarly, our results suggested that disclosing the evaluation results of irrational antibiotic prescriptions would also improve the level of antibiotic management by physicians and pharmacists. The publication of unreasonable prescriptions and the evaluation team’s suggestions for improvement served as valuable feedback for the continuous professional development of physicians and pharmacists. If there were any objections to the publication, a physician could report it to the evaluation expert panel and apply for its re-evaluation. As the last line of defence in our study, the expert group ensured the accuracy of the evaluation and improved the operational level of the evaluation team. This was borne out by the annual improvement in the initial response-to-evaluation consistency rate. More importantly, the change in antibiotic use did not affect disease treatment during the years when the system was implemented. There was no significant change in the rate of patient complaints regarding treatment outcomes.

However, there were limitations to the study. In this study, we were unable to evaluate whether patients without a prescription required antibiotic treatment. In addition, the system did not achieve the same results for antibiotic prescription management in the emergency department as it did in the outpatient department. There was a decrease in the proportion of single antibiotics prescribed in the emergency department, and there was a significant increase in the proportion of two-dose antibiotics prescribed (Table 1B). These results may be because most patients in the emergency department have an urgent condition, and the maximum antimicrobial treatment is administered to achieve a broader spectrum of antibiotic cover to save the lives of patients in critical situations. However, despite the implementation of evaluation systems, doctors did not avoid prescribing antibiotics when needed to prevent making a wrong prescription, which could cause a significant delay in the cure of disease. We will continue to study and improve the evaluation system.

## Conclusions

We established a monthly evaluation system of antibiotic prescription with three rounds of evaluation and a public feedback system. We verified the effectiveness of this system in clinical antibiotic use management, which improved the professional level of medical staff, including evaluation team members. At present, the system is running efficiently. However, due to the spread of COVID-19 in 2020, the number of patients decreased considerably. We only intercepted the system data from July 2016 to 2019 for verification to exclude the epidemic factors. More detailed research is needed to develop more effective management systems for antibiotic use.

## Data Availability

All relevant data are presented in the article/supplementary material; further inquiries can be directed to the corresponding author.
